# Clinical and MRI features of sacral insufficiency fractures after radiotherapy in patients with cervical cancer

**DOI:** 10.1186/s12905-022-01758-2

**Published:** 2022-05-13

**Authors:** Xi Zhong, Linqi Zhang, Tianfa Dong, Hui Mai, Bingui Lu, Lu Huang, Jiansheng Li

**Affiliations:** 1 Department of Medical Imaging, Affiliated Cancer Hospital & Institute of Guangzhou Medical University, Guangzhou, 510095 People’s Republic of China; 2 Department of Nuclear Medicine, Affiliated Cancer Hospital & Institute of Guangzhou Medical University, Guangzhou, 510095 People’s Republic of China; 3 Department of Radiology, The Third Affiliated Hospital of Guangzhou Medical University, Guangzhou, 510150 People’s Republic of China

**Keywords:** Insufficiency fracture, Magnetic resonance imaging, Cervical cancer, Radiotherapy

## Abstract

**Background:**

To determine the incidence, clinical and MRI features of sacral insufficiency fracture (SIF) after radiotherapy (RT) in patients with cervical cancer.

**Methods:**

Our study included 167 patients with cervical cancer after radiotherapy that underwent pelvic MRI for follow-up. MRIs included pre-enhanced T1-weighted, coronal fat-Suppressed T2-weighted (FS-T2W) and enhanced T1-weighted imaging. The clinical and MRI dates were reviewed. The gold standard of SIF was based on radiologic findings, clinical data and follow-up at least 12 months.

**Results:**

28 patients (10.8%) with 47 sites were diagnosed with SIFs, including 9 patients with unilateral SIF and 19 patients with bilateral SIFs. The median age was 60 years (range 41–72 years), and 89.3% (25/28) of patients were postmenopausal. 64.3% (18/28) of patients were symptomatic, and 53.6% of patients (15/28) had concomitant pelvic fractures. The median interval time from RT to SIFs was 10 months (range 3–34 months). For the lesion-wise analysis based on all MR images, all lesions were detected by visualizing bone marrow edema patterns, and fracture lines were detected in 64.6% (31/47) of SIFs. No soft-tissue tumors were founded. For each MRI sequence analysis, coronal FS-T2WI detected the most bone marrow edema pattern and fracture line than T1WI or enhanced T1WI.

**Conclusion:**

SIF is a common complication in cervical cancer after radiotherapy, which has some certain clinical and MRI features. Coronal FS-T2WI may be more useful to detect and characterize these fractures than other imaging sequences.

## Background

Radiotherapy (RT) is one of the main treatment methods for cervical cancer, and the late complications after radiotherapy have drawn more attention, included insufficiency fractures (IFs). IF has been considered as a common RT-induced complication, with the incidences ranged from 20 to 40% for cervical cancer, and the most common occurrence is sacrum, accounting for 75% of all Ifs [1–4]. The diagnosis of sacrum IF (SIF) after RT was challenging, because of SIF may not always show a fracture line, and SIFs without fracture line could be frequently misinterpreted as bone metastasis, which may result in unnecessary biopsy and aggressive radio-chemotherapy [5–10]. Hence, accurate diagnosis of SIF after RT is important and intractable issue in clinical practice.

Recently, the wide use of modern imaging modalities has improved the detection of SIFs, but the diagnostic sensitivity and specificity are still debatable. Bone scintigraphy (BS) is sensitive to detect SIF and the so-called “Honda sign” (H-sign) is well known as a characteristic sign, but this sign is often absent [11, 12]. Computed tomography (CT) has been shown to be specific in depicting fracture lines and osteosclerosis for SIF, but it may have limitations in sensitivity [13, 14].

MRI has been proved to be more sensitive to detect occult IFs than CT or BS, owing to the reveal of reactive bone marrow changes [13–15]. Previous literatures and our preliminary studies showed that MRI is extraordinary sensitive for revealing the reactive bone marrow edema associated with post-radiation IF and is useful for detecting soft-tissue mass, which may be valuable for identifying IF from bone metastasis, but MRI findings of insufficiency fractures may not always show a fracture line for SIF, with the disappearance of fracture line, the diagnosis of SIF is challenging [15–19]. Thus, detection of fracture line plays a great role in the diagnosis of SIF.

The coronal fat saturation-T2W (FS-T2W) imaging of sacrum has been recommended to detect fracture line in osteoporotic fracture and sacroiliitis, which shows superior value to other MRI sequences by increasing significant findings detection in 6.8% of patients [20]. To our knowledge, as for the radiation-induced SIF, the superiority of coronal FS-T2W to other MRI sequences in the detection of SIF has not been discussed. Therefore, we carried out this retrospective study to explore the incidence, clinical and MRI features of SIF after radiotherapy in patients with cervical cancer, and compared the ability of coronal FS-T2W imaging with other MRI sequences in the characterization of SIF after RT.

## Methods

### Patients

This retrospective study was approved by the institutional review board, and informed consent was not required. We retrospectively analyzed 167 cervical cancer patients received RT between July 2015 and December 2018. Pre-treatment and follow-up pelvic MRI was available for all patients; the median follow-up time was 45 months (range 25–72). The subject inclusion criteria as follows: (1) Pathology-proven cervical cancer, and received RT, (2) Pre-treatment pelvic MR showed no abnormal sign changes in the sacrum, (3) When emerging signal abnormality in sacrum was visualized after RT, one or more pelvic MRI and/or CT examination was performed during their at least 12 months follow-up. Exclusion criteria: (1) had sacrum metastasis, (2) had a history of pelvic trauma.

Finally, 28 patients (age range, 41–72 years; median age, 60 years) were identified as SIFs, whose clinical notes, symptoms and imaging findings were reviewed.

### Imaging acquisition

#### MR imaging

In all 28 subjects, pelvis MRI was performed by using a 1.5 T MR-scanner (Philips Achieva, Philips Healthcare, Best, The Netherlands). MR sequences included an axial T1-weighted spin-echo images (TR/TE, 496 ms/10 ms; matrix, 256 × 256; number of excitations [NEX], 2; echo-train length [ETL],1), an axial T2-weighted spin-echo images (TR/TE, 3500/100 ms; matrix, 512 × 256; NEX, 2; ETL,4) and an coronal fat-saturated T2-weighted images (TR/TE,2400/80 ms; SPAIR TR, 266 ms; matrix, 280 × 306; NEX, 2). Contrast-enhanced axial and sagittal T1-weighted images were also obtained by using a T1-weighted spin-echo sequence. For all scanning sequences, the field of view was 22–26 cm and the section thickness was 5 mm with a 2 mm interscan gap was performed.

#### Bone scintigraphy

Fifteen patients simultaneously underwent bone scintigraphy (BS) examination, BS studies were performed by using a SPECT/CT scanner (Philips, Netherlands, 4-slice diagnostic CT). The whole-body scan was performed 3 h after intravenous injection of 15~25 mCi 99mTc-MDP.

#### CT imaging

Eleven patients simultaneously underwent CT examination, imaging included the entire pelvis. CT studies were performed using a several MDCT scanners (64-MDCT scanners, Light Speed Series, GE Healthcare). All studies were performed with 120 kVP and milliampere values ranging from 200 to 300 mA.

### Image analysis

All images were analyzed at a diagnostic workstation (Advantage Windows, GE Healthcare, WI). The MRI, CT, and BS studies were analyzed separately in random order by two radiologists (8 and 10 years of experience in musculoskeletal imaging, respectively) in consensus. Radiologists were blinded to the patients’ identity and results of the clinical notes.

All patients underwent MRI and were evaluated for the absence or presence of a SIF and locations (unilateral or bilateral) of fractures. Furthermore, the absence or presence of a reactive bone marrow edema and fracture line in SIF was recorded. A bone marrow edema pattern was classified into three grades as follows [13]: (1) severe, the signal intensity on fat-saturated T2-weighted images was similar to that of spinal fluid or urine in the bladder, (2) moderate, bone marrow edema was visualized > 5 mm around the fracture line or had a diameter of > 10 mm if no fracture line was present and the signal intensity was lower than that of spinal fluid and urine in the bladder, (3) mild, bone marrow edema was visualized only along the fracture line (within 5 mm diameter) but not in the periphery. Fracture line was noted by linear low signal intensity on all MR sequences. The presence of bone marrow edema and fracture lines visualized on T1W, FS-T2W and enhanced T1W images were documented separately. Then, combined all MR images, the presence of bone marrow edema and fracture lines were documented. The presence and location of concomitant fractures in other sites (lumbar, pelvis and proximal femur) were also documented.

Presence of an “H-sign” was documented for 15 patients who had BS examination. “H-sign” is an H-shaped increase in areas of abnormal radiotracer uptake on the sacral body and both alae [11]. The presence of osteosclerosis and fracture line was documented for 11 patients who had CT examination.

### SIF reference standard

The conclusive diagnostic criterion was based on radiologic findings, clinical data and follow up at least 12 months. As described in previous studies, the diagnosis of SIF was based on the area of sclerosis without soft-tissue mass or better visualization fracture lines by follow-up CT, as well as areas of regressed bone marrow edema, stationary or fracture lines that were better visualized by MRI follow-up [13–15, 17].

## Statistical analysis

All the statistical tests were performed using SPSS Statistics 16.0 (SPSS Inc., Chicago, IL, USA) software package. Categorical data are expressed as numbers and frequency (%), and continuous data are expressed as median and range. Pearson chi-square test (or Fisher test) were performed for comparing the differences of detection sensitivity.

## Results

### Incidence

Of these167 cervical cancer patients after RT, we found 10.8% of the patients (28 patients) diagnosed with SIFs in the follow-up by using MRI.

### Patients’ clinical features

The median age was 60 years (range 41–72 years), and 85.7% (24/28) of patients aged ≥55 years. The clinical history, symptom, interval time from RT to MRI, associated fractures and additional imaging examination in 28 patients with SIF were showed in Table 1.

**Table 1:** The clinical history, RT project, symptom, interval time from RT to MRI, associated fractures and additional imaging examination in 28 patients with radiation-induced SIF detected by MRI

Patients	Postmenopausal	FIGO staging	RT project	Dose (Gy)	Symptom	Interval (Month)	Unilateral or bilateral SIF	Associated fractures	BS	CT
1	YES	IIA	Postoperative	50	Asymptomatic	10	Unilateral	NO	+	+
2	YES	IB1	Definitive	50	Asymptomatic	11	Bilateral	NO	+	NA
3	YES	IIB	Definitive	60	Asymptomatic	9	Unilateral	NO	+	NA
4	YES	IIA	Postoperative	56	Hip pain	6	Bilateral	NO	+	NA
5	YES	IIB	Definitive	50	Low back pain	13	Bilateral	Bilateral pubis, L5	+	NA
6	YES	IIIA	Definitive	50	Low back pain	13	Bilateral	Left acetabulum, L5	NA	+
7	YES	IB1	Definitive	50	Hip pain	14	Bilateral	L5	+	NA
8	YES	IIIB	Definitive	85	Hip pain	9	Bilateral	Bilateral pubis, L5	NA	+
9	YES	IB1	Definitive	60	Low back pain	4	Bilateral	Left ilium	+	NA
10	YES	IIA	Definitive	50	Asymptomatic	21	Bilateral	Left acetabulum	+	NA
11	YES	IV	Definitive	62	Asymptomatic	8	Unilateral	NO	NA	+
12	YES	IIB	Definitive	50	Asymptomatic	6	Unilateral	NO	NA	+
13	YES	IIB	Definitive	56	Hip pain	23	Bilateral	Right ilium	+	NA
14	YES	IVA	Definitive	63	Hip pain	34	Unilateral	Bilateral acetabulum	+	NA
15	YES	IVA	Definitive	90	Asymptomatic	5	Bilateral	Bilateral acetabulum	+	NA
16	YES	IIIB	Definitive	110	Hip pain	6	Unilateral	NO	+	NA
17	YES	IVA	Definitive	90	Hip pain	15	Bilateral	Left pubis	NA	+
18	YES	IIB	Definitive	50	Low back pain	7	Bilateral	NO	NA	+
19	YES	IIA	Definitive	50	Asymptomatic	3	Unilateral	NO	+	NA
20	YES	IIIB	Definitive	50	Hip pain	12	Bilateral	Bilateral ilium, pubis	+	NA
21	NO	IIIB	Postoperative	100	Asymptomatic	11	Unilateral	NO	NA	NA
22	YES	IIB	Definitive	68	Hip pain	7	Bilateral	Bilateral ilium	NA	+
23	YES	IIA	Definitive	62	Hip pain	11	Bilateral	Right acetabulum	NA	NA
24	YES	IIIB	Definitive	78	Hip pain	5	Bilateral	NO	NA	+
25	YES	IIB	Definitive	90	Asymptomatic	10	Bilateral	NO	+	NA
26	NO	IIB	Definitive	50	Hip pain	25	Bilateral	NO	NA	+
27	NO	IIIB	Definitive	110	Hip pain	5	Unilateral	Right ilium	NA	+
28	YES	IIIB	Definitive	50	Hip pain	12	Bilateral	Bilateral ilium	NA	NA

*FIGO* International Federation of Gynecology and Obstetrics, *RT* Radiotherapy, *BS* Bone scan, *NA* Not available

SIFs were frequently occurred in patients with a postmenopausal status, accounting for 89.3% (25/28) of patients. 89.3% (25/28) of patients accepted definitive RT, the median dose was 62 Gy (range 50–110 Gy); 3 patients accepted postoperative RT, the median dose was 56 Gy (range 50 – 100 Gy). The median interval time from RT to SIFs by MRI was 10 months (range 3–34 months), and the great majority of affected patients developed with SIFs within 2 years after RT (92.9%, 26 of 28 patients).

**Fig.1:**
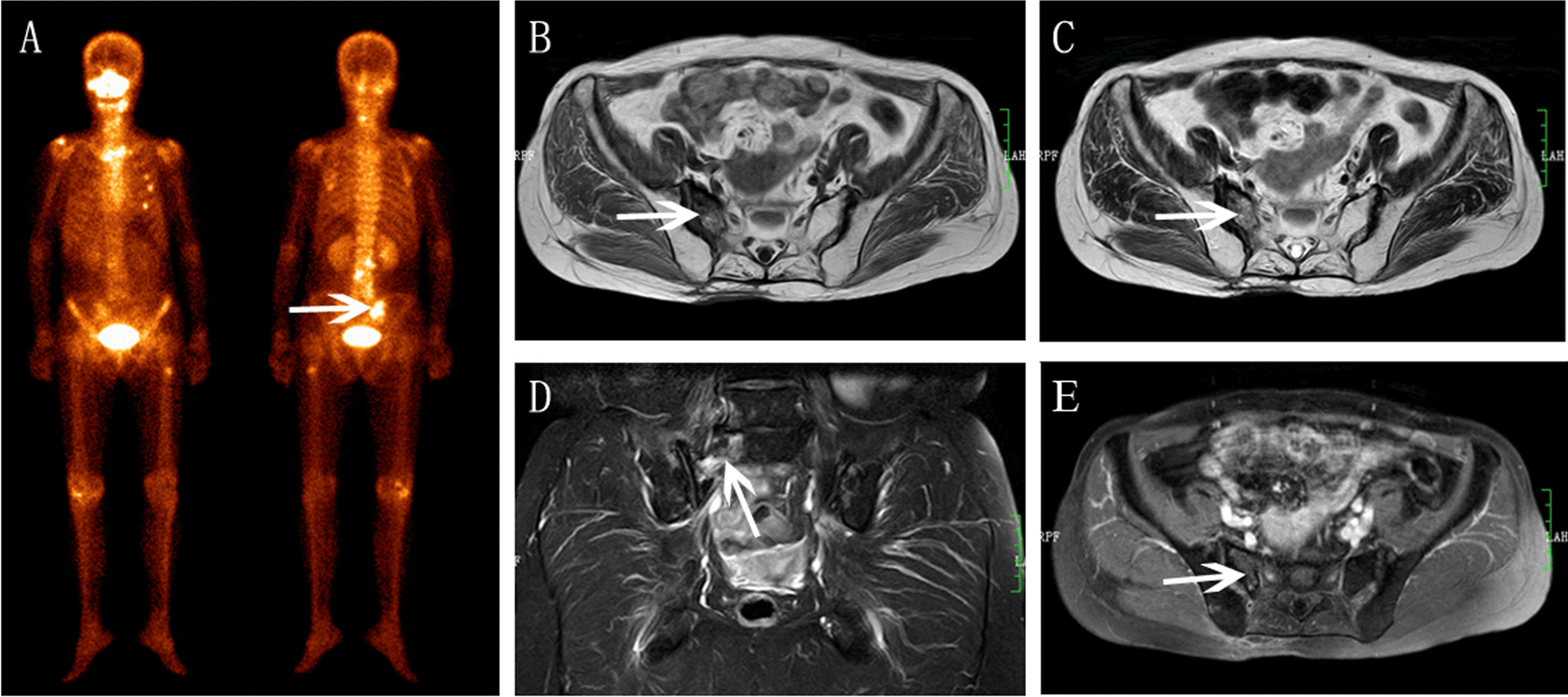
Right SIF in a 58-year-old woman with cervical cancer after radiotherapy. **A** BS showed right sacrum increased accumulation (arrow). **B** Axial T1WI showed hypointensity. **C** Axial T2WI showed moderate hyperintensity. **D** Coronal FS-T2WI showed severe bone marrow edema and diagonal fracture line (arrow). **E** Axial enhanced T1WI showed mild contrast enhancement

**Fig.2:**
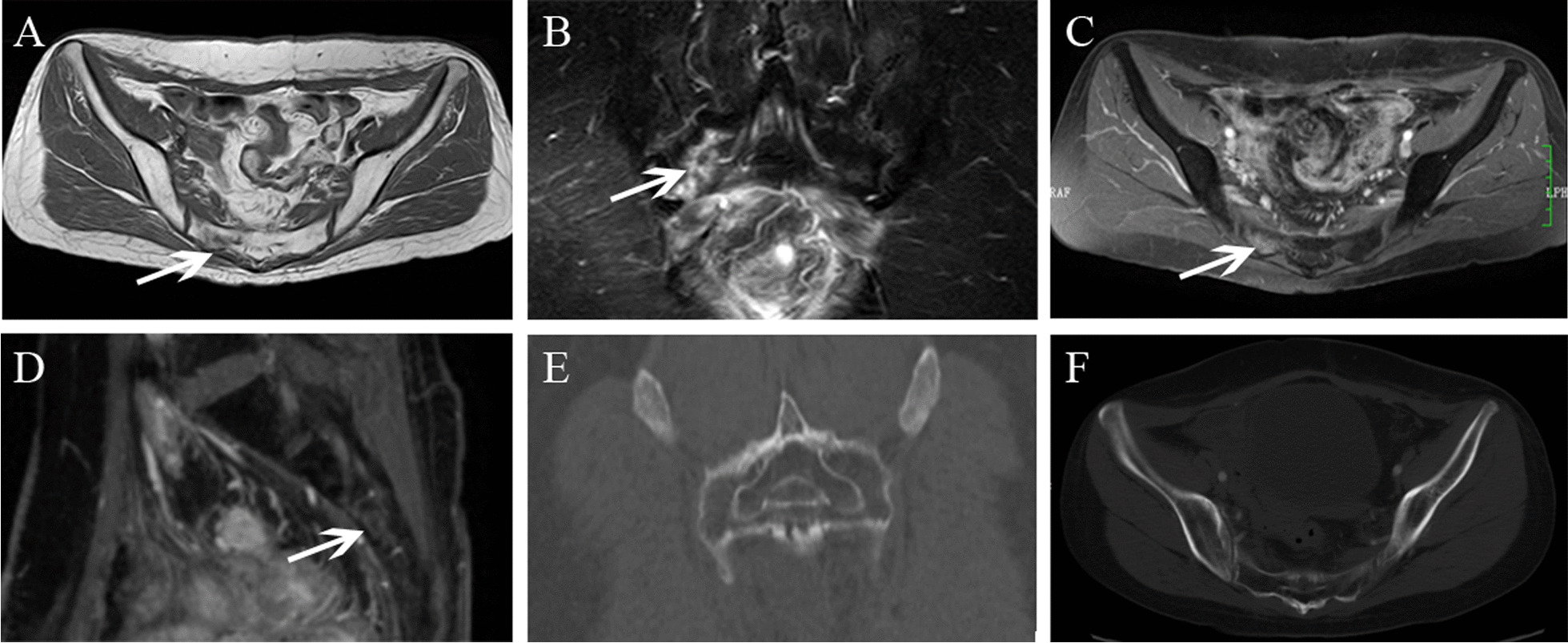
Right SIF in a 65-year-old woman with cervical cancer after radiotherapy. **A** Axial T1WI showed hypointensity. **B** Coronal FS-T2WI showed severe bone marrow edema with fracture line (arrow). **C**, **D** Axial and sagittal enhanced T1WI showed contrast enhancement. **E**, **F** Coronal and axial CT images showed no overt positive finding

**Fig.3:**
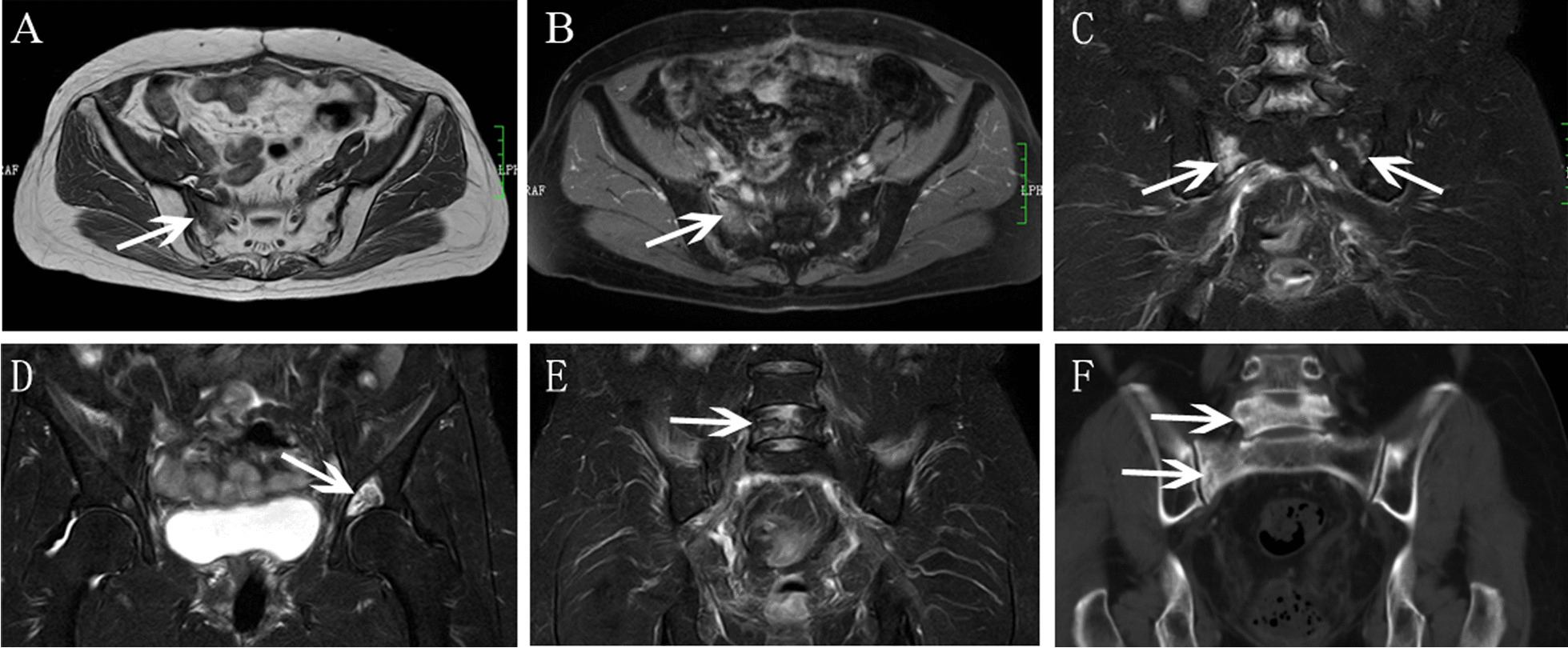
Bilateral SIFs had coexistent L5, acetabulum fractures in a 68-year-old woman with cervical cancer after radiotherapy. **A, B** Axial TWI and enhanced T1WI showed right sacrum abnormal signal (arrow). **C** Coronal FS-T2WI showed abnormal signal in bilateral sacrum, right sacrum showed severe bone marrow edema, and left sacrum showed mild bone marrow edema (arrow). **D, E** Coronal FS-T2WI showed acetabulum and L5 fractures. **F** Coronal CT image showed osteosclerosis change in L5 and right sacrum

**Fig.4:**
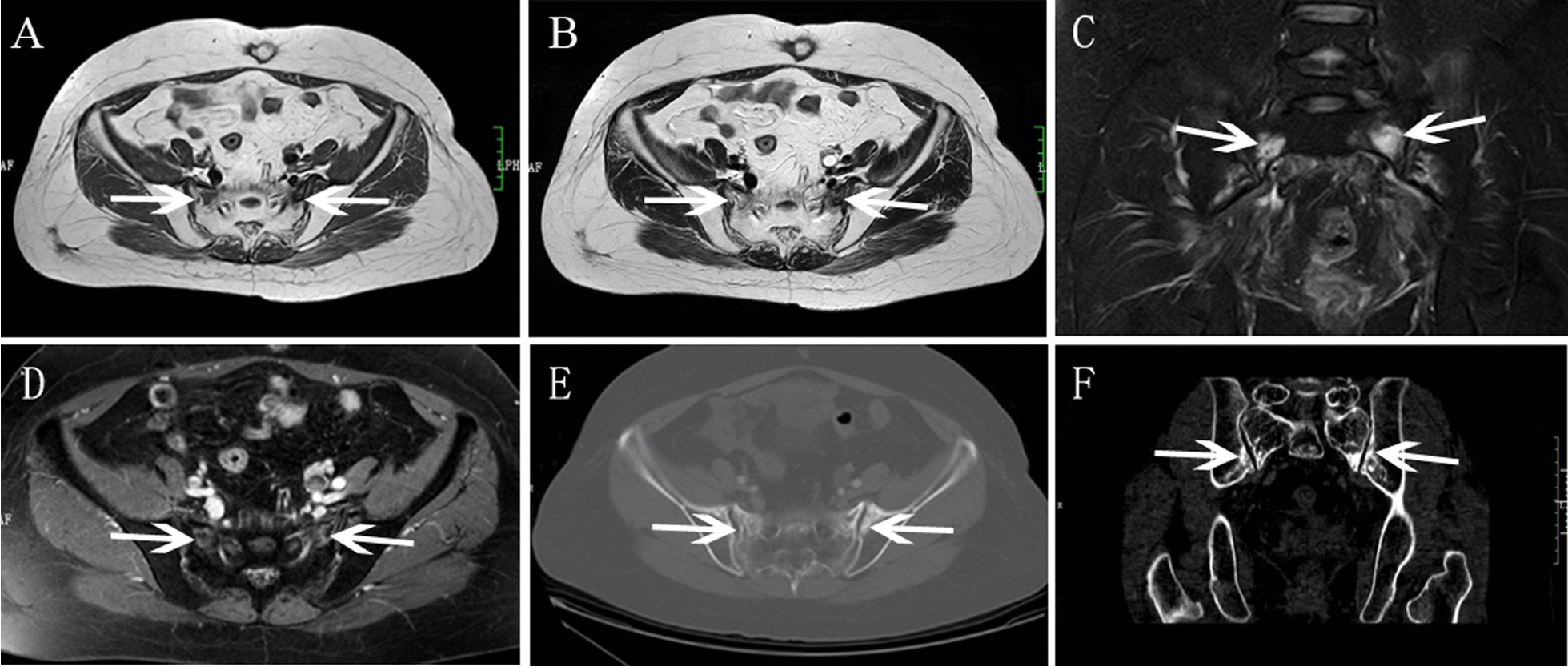
Bilateral SIFs had coexistent bilateral ilium fractures in a 70-year-old woman with cervical cancer after radiotherapy. **A, B** Axial T1WI and T2WI showed bilateral sacrum low signal (arrow). **C** Coronal FS-T2WI showed bilateral sacrum and ilium hyperintensity, bilateral sacrum with severe bone marrow edema. **D** Axial enhanced T1WI showed mild contrast enhancement in bilateral sacrum. **E, F**. Axial and coronal CT image showed osteosclerosis change

**Fig.5:**
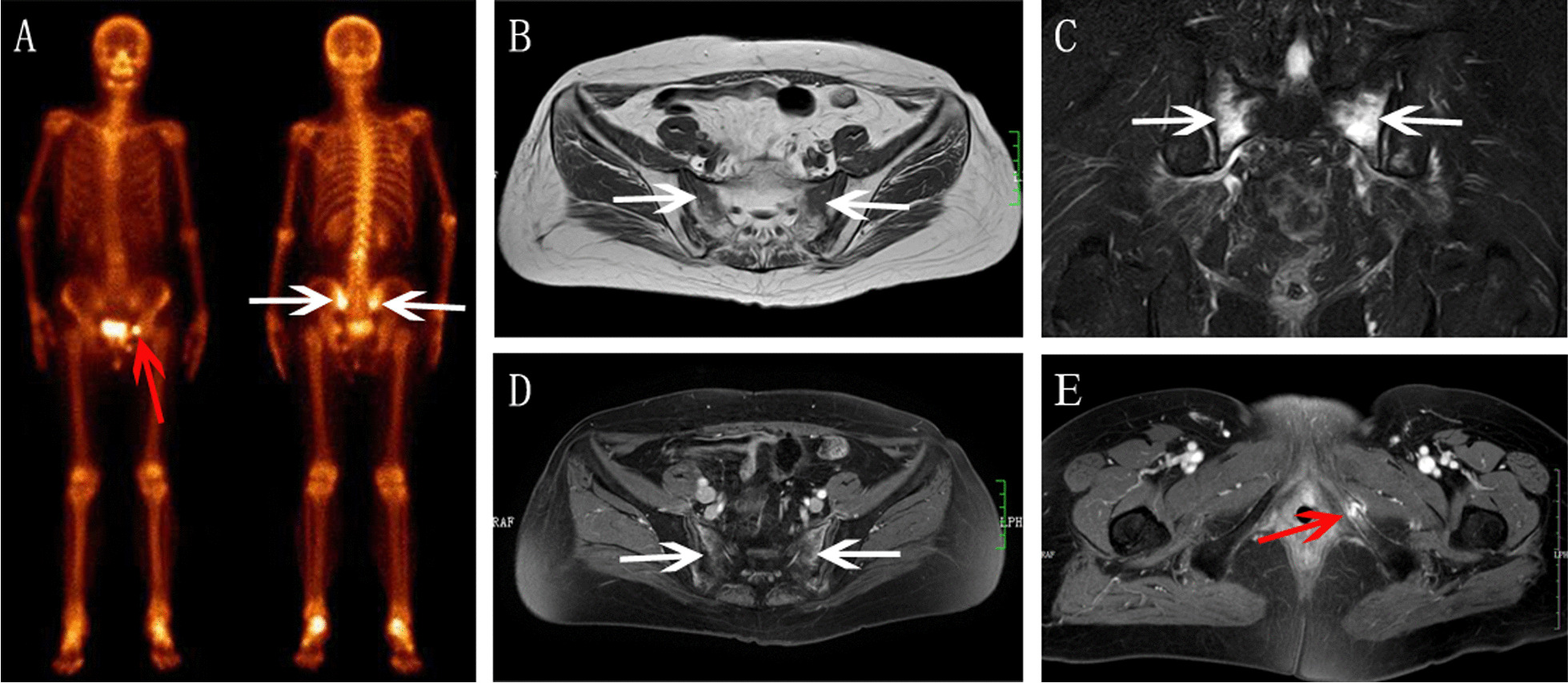
Bilateral SIFs had coexistent pubis fracture in a 63-year-old woman with cervical cancer after radiotherapy. **A** BS showed bilateral sacrum increased accumulation (white arrow) as an “H-sign”, and left pubis increased accumulation (red arrow). **B** Axial T1WI showed hypointensity. **C** Coronal FS-T2WI showed severe bone marrow edema. **D** Axial enhanced T1WI showed contrast enhancement in bilateral sacrum. **E** Axial enhanced T1WI showed enhancement with fracture line

Nine patients (31.0%) developed with unilateral SIFs (Figs. 1 and 2), and nineteen patients (69.0%) with bilateral SIFs (Figs. 3, 4 and 5). In total of 15 patients (53.6%) developed with concomitant fractures, include L5 fractures (14.3%, 4 patients) (Fig. 3), acetabulum fractures (17.8%, 5 patients) (Fig. 3), ilium fractures (21.4%, 6 patients) (Fig. 4) and pubis fractures (14.3%, 4 patients) (Fig. 5).

Eighteen patients (64.3%) had hip pain or low back pain, and ten patients were asymptomatic. 72.2% (13/18) of the symptomatic patients developed with concomitant fractures, but only 20% (2/10) of the asymptomatic patients with concomitant fractures.

### MRI findings

A total of 47 lesions were identified as SIFs in 28 patients (9 patients unilateral SIF, 19 patients bilateral SIFs) according to the reference standard. The detection rate of bone marrow edema and fracture line on MRI for the SIFs lesions were showed in Table 2.

**Table 2:** The detection rate of bone marrow edema and fracture line on MRI for the SIFs lesions (n=47)

	Bone marrow edema				Fracture line			
MRI sequences	T1WI	FS-T2WI	Enhanced T1WI	All images	T1WI	FS-T2WI	Enhanced T1WI	All images
Number	45	47	41	47	17	29	24	31
Percentage	95.7%	100%	87.2%	100%	36.2%	60.4%	51.1%	64.6%

For the lesion-wise analysis based on all MR images. All SIFs showed reactive bone marrow with hypointensity on pre-enhanced T1WI, hyperintensity on SPAIR-T2WI. According to the grading criterion, severe bone marrow edema patterns were found to be most frequently associated with SIFs (82.9%, 39/47), followed by moderate bone marrow edema (12.8%, 6/47), only two SIFs showed mild bone marrow edema. Fracture lines were also frequently founded in SIFs (Figs. 1, 2 and 4), accounting for 64.6% (31/47) of lesions, which was visualized as a linear low signal structure on either T1-weighted, SPAIR-T2 weighted and enhanced T1-weighted images. No soft-tissue tumor was founded in SIFs.

For individual MRI sequence analysis, coronal FS-T2WI detected more bone marrow edema pattern and fracture lines than pre-enhanced T1WI or enhanced T1WI (Table 2). The bone marrow edema pattern detection rates of coronal FS-T2WI was significantly higher than enhanced T1WI (100% vs 87.2% [41/47], P = 0.011), higher than pre-enhanced T1WI but without statistic difference (100% vs 95.7% [45/47], P = 0.153). Furthermore, the fracture lines detection rates of coronal FS-T2WI was significantly higher than pre-enhanced T1WI (60.4% [29/47] vs 36.2% [17/47], P=0.013), higher than enhanced T1WI but without statistic difference (60.4% [29/47] vs 51.1% [24/47], P=0.298).

### Additional BS and CT findings

15 patients with SIFs (5 patients, unilateral SIFs; 10 patients, bilateral SIFs) simultaneously underwent WBS, 8 patients (53.3%) showed the typical “H-sign” (Fig. 5). 11 patients simultaneously underwent CT (4 patients, unilateral SIFs; 7 patients, bilateral SIFs). Of the 18 SIFs, 15 lesions were involved osteosclerosis change (Figs. 3 and 4), fracture lines were showed in only 5 lesions, and other three lesions were invisible on CT (Fig. 2).

## Discussion

In the present study, we found that postradiotherapy SIF was a relatively common occurrence for patients with cervical cancer, and patients have some certain clinical characteristics, such as older ages with postmenopausal status, developed with SIFs within 2 years after RT, involved bilateral sacrum, concomitant fractures. MRI, especially coronal FS-T2W imaging was useful to detect and character SIFs.

The sacrum is the initial site of fracture and then lead to increased stress on other sites of the pelvis. The actual incidence of RT-induced SIFs is unclear, the incidence in our study was 10.8%, which was similar with previous studies [21–24]. In our study, almost 53.6% of patients had concomitant fractures, suggested that identification of SIFs should arouse clinical suspicion of fractures in other sites. However, SIFs are most frequently associated with pubis fractures, with a reported coincidence of 78% [24]. We found that only 14.3% patients had coexistent pubis fractures, it was lower than that of the presences of fracture in L5 (14.3%), acetabulum (17.8%), ilium (21.4%).

The clinical presentation of SIF is vague and non-specific. We observed almost two-thirds of SIFs were clinically symptomatic, the incidence was higher than a study reported about one third [21]. First, this may be most likely explainable by the fact that 53.6% of patients with concomitant fractures were included, and 72.2% of the symptomatic patients developed with concomitant fractures. Multiple site fractures may be contributed to some patients with pain [9]. Second, 82.9% of SIFs presented severe bone marrow edema patterns in present study, which was the same as Blomlie et al. [25] revealed, larger lesions (> 1 cm^2^) on MRI tended to be more likely painful.

Older patients with postmenopausal status after RT are more susceptible to the development of IF [1, 2, 9]. We found that SIFs frequently occurred in postmenopausal patients, and the median age was 60 years. Based on our results, most of affected patients developed with SIFs within 2 years, and the median interval time from RT to SIFs was 10 months, which is almost similar with previous studies with a median interval time between 8 and 14 months [1, 6, 21]. Supporting a previous study demonstrated that 88.9% of patients with RT-induced SIFs were bilateral [19], we observed over two-third of SIFs have arosen bilaterally.

Although SIFs are rarely life-threatening, they should be deserved special attention because they can influence the quality of life [26]. Only 20–38% of SIF could be identified on plain films [27–29]. Recently, modern imaging modalities during follow-up have been applied in the detection and characterization of SIFs. Both BS and CT have some limitations, BS is one of the most sensitive imaging modalities for detecting SIF, but the “H-sign” is often absent [11]. In our study, only 53.3% (8/15) of patients who had additional BS showed the typical “H-sign”. CT is useful to detect fracture lines, which has been shown to be less sensitive to detect SIFs than MRI, with a recorded sensitivity between 60 and 75% [13, 14].

MRI is an alternative technique due to its high soft-tissue contrast, multiplanar imaging, and avoidance of ionizing radiation. MRI is one of the most sensitive imaging techniques to detect RT-induced IF by visualizing the bone marrow edema. Cabarrus et al. [13], showed that the overall sensitivity of MRI was significantly higher than CT (100 vs 74.6%), although the sensitivity for detecting fracture lines was similar (93.3% for MRI vs 89.7% for CT). We found all SIFs showed bone marrow edema, and fracture lines were visualized in 64.6% of lesions. Furthermore, MRI is also helpful to differentiate SIF from the bone metastasis, because MRI is very useful to identify the soft-tissue component, the absence of focal or discrete soft-tissue mass around fracture sites is an important sign for distinguishing SIF from malignancy [15–19]. In present study, no soft-tissue tumor was detected in fracture sites.

The FS-T2W imaging is especially sensitive for visualizing early bone marrow edema, and FS-T2W imaging is recommended to be included in suspected fracture cases [30]. Gupta et al. [30] demonstrated that coronal STIR sequence had additional value to the L-spine MRI by increasing significant findings detection in 6.8% of patients, including SIF or sacroiliitis [20]. The advantage of our study was that both coronal FS T2-weighted and gadolinium-enhanced T1-weighted imaging were performed for all patients. We found coronal FS-T2WI was more sensitive to detect both bone marrow edema and fracture lines than that of T1WI or enhanced T1WI. In addition, this study revealed that T1WI detected the least fracture lines because of bone marrow edema pattern was also hypointensity, and enhanced T1WI detected least bone marrow edema due to some SIFs with no or mild enhancement, which may be difficult to identify.

There were several limitations to our study. First, our study was retrospectively performed in a single-institution. Second, the follow-up period was inconsistent (range, 25–72 months), which might have resulted in underestimation of the true prevalence of SIF. Third, only a small number of patients undergone simultaneous BS or CT examinations, we were unable to compare the diagnostic ability of MRI with BS or CT. Fourth, none of the IF lesion was diagnosis based on histopathology, as a pathologic diagnosis was generally impractical and actually unnecessary.

## Conclusion

SIF is a common complication for patients with cervical cancer after radiotherapy, which has some certain clinical and MRI features. MRI, especially coronal FS-T2WI may be more useful to detect and characterize these fractures than other imaging sequences. Knowing well these features help to prevent confusion with metastatic disease and inappropriate treatment.

## Data Availability

The datasets used and/or analyzed during the current study available from the corresponding author on reasonable request.

## Abbreviations

RT: Radiotherapy
IF: Insufficiency fracture
SIF: Sacral insufficiency fracture
MRI: Magnetic resonance imaging
CT: Computed tomography
BS: Bone scintigraphy
FS-T2W: Fat-suppressed T2-weighted
